# Anxiety, Depression, and Care Barriers in Adults With Intellectual and Developmental Disabilities

**DOI:** 10.1001/jamanetworkopen.2025.60205

**Published:** 2026-02-20

**Authors:** Anthony R. Osuna, Jae Kennedy, Chuan Zhou, Dimitri A. Christakis

**Affiliations:** 1 Department of Pediatrics, University of Washington School of Medicine, Seattle; 2 Center for Child Health, Behavior, and Development, Seattle Children’s Research Institute, Seattle, Washington; 3 Department of Community Health, College of Medicine, Washington State University, Pullman; 4 Center for Clinical and Translational Research, Seattle Children’s Research Institute, Seattle, Washington; 5 Special Olympics International, Washington, DC; 6 Editor-in-Chief, *JAMA Pediatrics*

## Abstract

**Importance:**

Adults with intellectual and developmental disabilities (IDDs) experience relatively high rates of psychiatric conditions; however, national estimates of mental health disparities and barriers to access to care remain limited.

**Objective:**

To examine the prevalence of anxiety and depression, mental health treatment, and cost-related barriers among adults with IDDs compared with those without functional limitations.

**Design, Setting, and Participants:**

This cross-sectional study used pooled US National Health Interview Survey data collected from January 1, 2021, to December 31, 2023. The analytic sample included 44 478 adults 18 years or older, representing 134.3 million US adults. Adults with IDDs were identified using Washington Group Short Set criteria, indicating significant difficulty with cognition, communication, or self-care beginning before 22 years of age. The sample included 796 adults with IDDs (weighted population, 2.9 million) and 43 682 adults without functional limitations (weighted population, 131.4 million). Data were analyzed from August 18 to December 18, 2025.

**Exposure:**

US National Health Interview Survey.

**Main Outcomes and Measures:**

Outcomes included self-reported diagnoses of anxiety and depression, symptom frequency and severity, psychiatric medication use, receipt of past-year counseling or therapy, and cost-related barriers to mental health care. Weighted prevalence estimates and multivariable logistic regression models were used to examine associations between IDD status and outcomes, adjusting for demographic and socioeconomic covariates. Sensitivity analyses incorporated multiple imputation and propensity score weighting.

**Results:**

Among 44 478 adults included in the analysis (estimated 52.8% male; mean [SD] age, 48.6 [16.5] year), the 796 adults with IDDs were younger and more likely to have a lower income and public insurance coverage. Diagnosed anxiety occurred in an estimated 57.2% of adults with IDDs vs 10.6% of adults without limitations (adjusted odds ratio [AOR], 9.41; 95% CI, 7.71-11.49), and diagnosed depression occurred in an estimated 57.1% of adults with IDDs vs 9.9% of adults without limitations (AOR, 9.78; 95% CI, 8.08-11.83). Adults with IDDs also reported more common daily anxiety (50.8% vs 7.9%; AOR, 9.72; 95% CI, 8.08-11.71) and daily depression (25.6% vs 1.4%; AOR, 17.73; 95% CI, 13.84-22.72), use of prescribed medications for anxiety (41.7% vs 8.6%; AOR, 7.02; 95% CI, 5.73-8.60) and depression (38.2% vs 6.0%; AOR, 8.88; 95% CI, 7.28-10.83), and past-year use of counseling or therapy (41.4% vs 9.0%; AOR, 5.83; 95% CI, 4.78-7.11).

**Conclusions and Relevance:**

In this cross-sectional study, US adults with IDDs experienced substantially higher rates of mental health conditions, treatment use, and cost-related barriers compared with those without functional impairments. These findings highlight critical gaps for accessible, affordable, and disability-informed mental health services and policy reforms to address systemic inequities.

## Key Points

**Question:**

Are anxiety, depression, and barriers to mental health care more prevalent among adults with intellectual and developmental disabilities (IDDs) than those without functional limitations?

**Findings:**

In this cross-sectional study of 44 478 US adults from the 2021, 2022, and 2023 National Health Interview Surveys, adults with IDDs had a higher prevalence of diagnosed anxiety and depression, more frequent and severe symptoms, greater psychiatric medication use, and more cost-related barriers to counseling or therapy than adults without functional limitations.

**Meaning:**

These findings suggest that adults with IDDs experience significant mental health disparities and barriers to care, indicating a need for disability-informed mental health services and policies.

## Teaser

This cross-sectional study uses data from the National Health Interview Surveys to assess the prevalence and severity of anxiety and depression, use of mental health care, and cost-related barriers to care among US adults with and without intellectual and developmental disabilities (IDDs).

## Introduction

Intellectual and developmental disabilities (IDDs) are lifelong conditions that begin in childhood and involve significant difficulties with cognitive abilities and daily life skills.^1^ IDD prevalence estimates vary but suggest that 1.0% to 8.5% of the US population^2,3^ and 1.0% to 3.0% globally^4,5^ have a developmental disability. People with IDDs face pronounced health challenges, including high rates of chronic medical conditions, unmet health care needs, and barriers to accessing high-quality care.^6^ Although awareness of these disparities is increasing, many people with IDDs remain uncounted in national health datasets, making it challenging to monitor population-level trends.^7^ To address these gaps, improved public health surveillance and standardized, inclusive methods for identifying people with IDDs in national data sources are urgently needed.^8^

Disability research studies have documented consistently elevated rates of psychiatric diagnoses among people with IDDs^4^ but are limited in scope and have not captured the broader range of mental health and health care disparities this population faces. National Core Indicators survey data suggest that as many as 45% of adults with IDDs have at least 1 mental health condition,^9^ and Medicaid claims indicate that more than half of beneficiaries with IDDs have a psychiatric diagnosis.^10^ Among individuals with IDDs receiving state-funded services in Virginia, 29% had a mood disorder and 24% had an anxiety disorder,^11^ rates that exceed those in the general US adult population, where approximately 8.3% experience depression^12^ and 19.1% have an anxiety disorder.^13^ Clinical data from pediatric inpatient settings underscore these disparities, showing that children with developmental disabilities were more likely than peers without developmental disabilities to have Medicaid insurance, be readmitted, receive psychotropic medication for aggression or anxiety, and experience physical restraint during hospitalization.^14^

Structural barriers contribute to these disparities. Adults with IDDs often encounter inadequate transportation, long wait times, and inaccessible telehealth platforms that delay or interrupt care.^14^ Compounding these issues are clinician-level barriers, such as insufficient training in disability-inclusive care, communication difficulties, and persistent stigmatizing attitudes toward people with IDDs.^15^ These issues are particularly pronounced for individuals from marginalized or underresourced communities, who experience even higher rates of delayed care, reduced access, and discrimination in health care settings.^16^

Limited national surveillance data hamper efforts to quantify these inequities. Until recently, few large-scale surveys included measures that allowed identification of adults with IDDs. In 2019, the National Health Interview Survey (NHIS), a longstanding federal health surveillance tool, was redesigned to include the Washington Group Short Set (WG-SS) on Functioning, which assesses difficulty in areas such as cognition, communication, and self-care. Although not diagnostic, these items allow researchers to identify likely cases of IDDs using proxy criteria.^17^

The NHIS also collects detailed information on mental health symptoms, psychological distress, service use, and access barriers, making it a valuable resource for studying psychiatric experiences. The objective of this study was to quantify differences in the prevalence and severity of anxiety and depression, mental health treatment use, and cost-related barriers to care between US adults with likely IDDs and adults without functional limitations using nationally representative data from the NHIS. We hypothesized that, compared with adults without functional impairments, and after adjustment for demographic and socioeconomic covariates, adults with IDDs would experience significantly higher rates of anxiety and depression, greater symptom severity, greater use of psychiatric medications and therapy, and greater cost-related barriers to mental health care.

## Methods

### Data Source and Study Sample

This cross-sectional study used pooled data from the NHIS Sample Adult Surveys collected from January 1, 2021, to December 31, 2023. The NHIS is a nationally representative cross-sectional survey conducted annually by the US National Center for Health Statistics. It collects data on health status, health care access, and health behaviors from the noninstitutionalized civilian US population using a multistage stratified sampling design. In each participating household, 1 adult 18 years or older is randomly selected to complete the Sample Adult questionnaire, typically via self-report, although proxy responses are permitted by NHIS protocol when respondents are unable to participate directly due to cognitive, physical, or communication limitations. The survey includes information on physical and mental health, use of health care services, chronic conditions, and functional limitations. Response rates for the NHIS Sample Adult Surveys were 50.9% in 2021, 47.7% in 2022, and 47.0% in 2023.^18^ Because this study used publicly available, deidentified data, it was deemed exempt from Institutional Review Board review by the Common Rule due the use of deidentifed data. The National Center for Health Statistics obtained verbal informed consent as part of the NHIS data collection process. This study followed the Strengthening the Reporting of Observational Studies in Epidemiology (STROBE) reporting guideline.

### Disability Group Classification

Respondents were identified using the WG-SS of disability questions as proxy criteria, given the absence of formal diagnostic data in the NHIS. Respondents were classified as having an IDD if they selected the responses “a lot of difficulty” or “cannot do at all” for at least 1 of 3 functional domains (cognition, self-care, or communication) and indicated the limitation began before 22 years of age, consistent with prior NHIS-based research.^17^ The comparison group consisted of individuals with no difficulty in any WG-SS domain. Respondents with vision, hearing, or mobility limitations were included in the analysis but not in the operational definition of IDD. Those who became limited in cognition, self-care, or communication at 22 years or older were excluded to maintain alignment with developmental onset criteria.

### Demographic and Socioeconomic Characteristics

Race and ethnicity were self-reported by participants using predefined NHIS survey response categories and collected by trained NHIS interviewers as American Indian or Alaska Native, Asian, Black or African American, Hispanic or Latino, White, multiracial, or other race or ethnicity (defined as mixed American Indian or Alaska Native and other single or multiple race categories in the NHIS coding). Race and ethnicity were included to capture structural and social factors that may influence mental health outcomes and service access. These data are presented in Table 1.

**Table 1.** Demographic and Socioeconomic Characteristics of the Study Population^a^

Characteristic	Adults with IDDs		Adults with no limitations		*P* value^b^
	Sample No.	Estimated No. in millions (weighted %)	Sample No.	Estimated No. in millions (weighted %)	
All participants	796	8.6 (100)	43 682	394.2 (100)	NA
Survey years					
2021	258	2.4 (28.3)	15 105	132.5 (33.6)	.006
2022	242	2.8 (32.5)	13 986	130.7 (33.2)	
2023	296	3.4 (39.2)	14 591	131.0 (33.2)	
Age, y					
22-34	361	4.8 (55.4)	10 604	117.1 (29.7)	<.001
35-44	147	1.4 (16.6)	9449	88.4 (22.4)	
45-54	111	1.0 (12.2)	7100	69.3 (17.6)	
55-64	101	0.8 (9.7)	7451	61.7 (15.6)	
65-74	45	0.3 (3.6)	6189	41.0 (10.4)	
75-85	31	0.2 (2.5)	2889	16.7 (4.2)	
Sex					
Male	362	4.4 (50.5)	20 642	199.1 (50.5%)	>.99
Female	434	4.3 (49.5)	23 034	195.0 (49.5)	
Race and ethnicity					
American Indian or Alaska Native	15	0.2 (1.8)	208	2.0 (0.5)	
Asian	22	0.2 (2.9)	3459	30.7 (7.8)	<.001
Black or African American	80	1.0 (12.1)	4858	47.2 (12.0)	
Hispanic or Latino	115	1.3 (15.0)	7006	73.5 (18.6)	
White	539	5.6 (65.0)	27 359	233.6 (59.3)	
Multiracial	7	0.04 (0.5)	228	2.0 (0.5)	
Other^c^	18	0.2 (2.7)	564	5.2 (1.3)	
US region					
Northeast	120	1.4 (15.8)	7584	74.5 (18.9)	.20
Midwest	166	1.6 (19.1)	9110	78.6 (19.9)	
South	297	3.5 (40.8)	15 581	146.7 (37.2)	
West	213	2.1 (24.2)	11 407	94.4 (23.9)	
Community location					
Urban or suburban	652	7.3 (84.7)	38 236	349.0 (88.5)	.003
Rural	144	1.3 (15.3)	5446	45.2 (11.5)	
Family income below federal poverty level	193	1.7 (19.9)	3176	29.2 (7.4)	<.001
Health insurance coverage					
Private	323	3.4 (39.7)	33 330	293.6 (74.7)	<.001
Public	406	4.5 (52.9)	6410	57.3 (14.6)	
None	61	0.6 (7.3)	3815	42.0 (10.7)	

Abbreviations: IDD, intellectual and developmental disability; NA, not applicable; NHIS, National Health Interview Survey.

^a^ Source: 2021, 2022, and 2023 National Health Interview Surveys, Sample Adult Files (N = 44 478).

^b^ Based on Pearson χ^2^ test with Rao-Scott adjustment for survey design.

^c^ Includes respondents coded by NHIS as mixed American Indian or Alaska Native or other single or multiple race.

### Mental Health Outcomes, Service Use, and Access

Participants reported whether a health care professional had ever diagnosed them with an anxiety disorder or depression and whether they were currently taking prescription medication for either condition. Symptom burden was assessed by asking how often they felt anxious or depressed (ranging from never to daily) and the severity of their most recent symptoms (ranging from a little to a lot). Use of mental health care services was measured by whether they had received counseling or therapy from a mental health professional in the past 12 months. Two additional items assessed access barriers: whether there was any time in the past year when they needed counseling or therapy but did not receive it due to cost, and whether they delayed seeking services for the same reason.

### Statistical Analysis

Data were analyzed from August 18 to December 18, 2025. Bivariate comparisons were conducted using Wald χ^2^ tests. Adjusted odds ratios (AORs) and 95% CIs were estimated using design-based logistic regression models. Two-sided *P* < .05 indicated statistical significance. All models controlled for age group, sex, race and ethnicity, region, urban-rural status, income-to-poverty ratio, and health insurance type. Missing data were handled using complete-case analysis. Analyses were conducted using R, version 4.5.2 (R Foundation for Statistical Computing) and the survey package (version 4.4) for complex survey weighting and variance estimation, which implements design-based methods for stratified, clustered, and weighted samples.^19,20,21^

Survey year was included as a covariate to account for temporal variation in mental health service use and access patterns during the COVID-19 period. Missingness was low across outcomes and covariates (<3% for all variables), and primary analyses used complete-case estimation. Sensitivity analyses were conducted using multiple imputation with chained equations followed by propensity score–based weighting. Results of these sensitivity analyses are presented in eTable 1 in Supplement 1.

## Results

### Demographic Characteristics

The analytic sample consisted of 44 478 US adults, including 796 adults with IDDs and 43 682 adults without functional limitations (Table 1). The mean (SD) age was 48.6 (16.5) years; an estimated 52.8% were female and 47.2% were male. In terms of race and ethnicity, 0.5% were American Indian or Alaska Native; 7.8%, Asian; 11.1%, Black or African American; 16.0%, Hispanic or Latino; 62.7%, White; 1.8%, multiracial or other race or ethnicity. Compared with their peers without disabilities, adults with IDDs were significantly more likely to live in rural areas, have family incomes below the federal poverty level, and rely on public health insurance. The sociodemographic and socioeconomic characteristics of the 2 groups, along with each of the survey years, were used as statistical controls in the logistic models in Table 2. Supplemental analyses (available on request) confirmed that mental health and health care disparities did not differ by survey year.

**Table 2.** Prevalence and Odds of Anxiety Disorder or Depression Diagnosis, Pharmacologic Treatments, Frequency and Severity of Symptoms, and Use of and Access to Mental Health Services^a^

Variable	Adults with IDDs (n = 796)		Adults with no limitations (n = 43 682)		IDDs vs no limitations	
	Sample No.	Estimated No. in millions (weighted %)	Sample No.	Estimated No. in millions (weighted %)	AOR (95% CI)	*P* value
Anxiety						
Diagnosed with anxiety disorder	467	4.9 (57.2)	4721	41.9 (10.6)	9.41 (7.71-11.49)	<.001
Taking medication for anxiety	346	3.5 (41.7)	3829	33.0 (8.6)	7.02 (5.73-8.60)	
Feeling anxious daily	405	4.2 (50.8)	3320	30.5 (7.9)	9.72 (8.08-11,71)	
Feeling a lot of anxiety	274	2.8 (32.5)	2133	18.9 (4.8)	8.08 (6.65-9.81)	
Depression						
Diagnosed with depression	485	4.9 (57.1)	4574	39.0 (9.9)	9.78 (8.08-11.83)	<.001
Taking medication for depression	324	3.2 (38.2)	2772	23.0 (6.0)	8.88 (7.28-10.83)	
Feeling depressed daily	210	2.1 (25.6)	602	5.2 (1.4)	17.73 (13.84-22.72)	
Feeling a lot of depression	254	2.5 (29.0)	1425	12.4 (3.2)	9.07 (7.38-11.15)	
Mental health services						
Received counseling or therapy in past 12 mo	334	3.5 (41.4)	4092	34.7 (9.0)	5.83 (4.78-7.11)	<.001
Needed but delayed counseling or therapy due to cost	147	1.5 (17.9)	1496	13.6 (3.5)	5.03 (3.97-6.39)	
Needed but did not get counseling or therapy due to cost	152	1.6 (19.2)	1365	12.5 (3.2)	5.57 (4.37-7.10)	

Abbreviations: AOR, adjusted odds ratio; IDD, intellectual and developmental disability.

^a^ Source: 2021, 2022, and 2023 National Health Interview Survey, Sample Adult Files (N = 44 478). Models were adjusted for survey year, age group, sex, race and ethnicity, US region, community population size, income, and health insurance coverage. All models were weighted with survey sampling weights.

### Prevalence and Severity of Mental Health Conditions

Table 2 shows consistently higher prevalence of anxiety and depression among adults with IDDs, compared with adults without functional limitations. Among the estimated 8.61 million adults with IDDs, 57.2% reported an anxiety diagnosis compared with 10.6% of the estimated 394.2 million adults without limitations (AOR, 9.41; 95% CI, 7.71-11.49). Likewise, 57.1% adults with IDDs reported a depression diagnosis compared with 9.9% without limitations (AOR, 9.78; 95% CI, 8.08-11.83).

Symptom burden was also elevated among adults with IDDs; 50.8% reported daily anxiety vs 7.9% of adults without limitations (AOR, 9.72; 95% CI, 8.08-11.71) and 25.6% reported daily depression vs 1.4% of adults without limitations (AOR, 17.73; 95% CI, 13.84-22.72). Symptom severity was also higher among adults with IDDs; 32.5% reported feeling a lot of anxiety vs 4.8% of adults without limitations (AOR, 8.08; 95% CI, 6.65-9.81) and 29.0% reported feeling a lot of depression compared with 3.2% adults without limitations (AOR, 9.07; 95% CI, 7.38-11.15).

### Mental Health Treatment and Access Barriers

Table 2 also shows that adults with IDDs were much more likely to receive pharmacologic treatment for anxiety and depression, as well as counseling or therapy: 41.7% reported using medication for anxiety compared with 8.6% of adults without limitations (AOR, 7.02; 95% CI, 5.73-8.60), 38.2% reported taking medication for depression vs 6.0% of adults without limitations (AOR, 8.88; 95% CI, 7.28-10.83), and 41.4% engaged in therapy or counseling within the past year compared with 9.0% of adults without limitations (AOR, 5.83; 95% CI, 4.78-7.11).

The higher incidence of mental health problems and use of mental health treatments was also associated with more access barriers among adults with IDDs: approximately 17.9% reported delaying needed therapy or counseling due to cost compared with 3.53% of adults without limitations (AOR, 5.03; 95% CI, 3.97-6.39). In addition, 9.2% of adults with IDDs reported not getting needed therapy or counseling due to cost, compared with 3.2% of adults without limitations (AOR, 5.57; 95% CI, 4.37-7.10).

## Discussion

This cross-sectional study provides nationally representative evidence of pronounced disparities in mental health outcomes, use of services, and access barriers to psychiatric care between US adults with likely IDDs and those without impairments. Adults with IDDs were more than 9 times more likely to report a diagnosis of anxiety or depression and experienced significantly higher symptom burden. These rates align with previous studies documenting elevated psychiatric conditions, trauma, and mental health access among individuals with IDDs.^9,10,11,22,23^ While these findings reinforce prior literature, this study adds important national-level evidence to a growing body of research calling for urgent action in mental health care for people with IDDs.

The elevated burden of depression and anxiety among individuals with IDDs has serious implications for well-being and functioning. These conditions are known to reduce quality of life, interfere with social participation, and increase suicide risk.^24^ For people with IDDs, these impacts are often intensified by additional barriers such as diagnostic overshadowing, limited access to tailored services, and a lack of developmentally appropriate screening and intervention tools.^25,26,27^ Mental health diagnoses are closely associated with suicidality,^28^ and adults with disabilities are significantly more likely to report suicidal thoughts and behaviors compared with those without disabilities.^29^ These findings highlight the urgent need for accessible, developmentally appropriate, and disability-informed mental health services across the lifespan.

Although medication use was higher among adults with IDDs, use of counseling or therapy did not appear proportionate to the magnitude of symptom burden or need. In the full IDD sample, approximately 40% of adults reported receiving counseling or therapy in the past year, despite a substantially higher prevalence of diagnosed anxiety and depression and more frequent and severe symptoms compared with adults without functional limitations. Historically, mental health care for individuals with IDDs has relied heavily on pharmacologic approaches,^30^ contributing to a lack of investment in adapted psychotherapeutic approaches. Evidence-based therapies such as cognitive behavioral therapy have shown promise when adapted for people with IDDs,^31^ but access remains limited.

Importantly, these treatment patterns likely reflect multiple mechanisms beyond overreliance on pharmacologic treatment alone. Adults with IDDs may have greater overall contact with the health care system due to higher rates of chronic medical conditions and disability-related support needs, which may increase opportunities for mental health diagnosis and medication prescribing during routine medical encounters. In contrast, structural barriers, including limited availability of clinicians trained in disability-informed psychotherapy, long wait times, transportation challenges, and difficulties navigating behavioral health systems, may restrict access to counseling or therapy even when clinical need is high.^27^ Additional research and resources are urgently needed to expand access to developmentally appropriate, evidence-based therapy services for this underserved population.^32^

Cost also emerged as a significant barrier: adults with IDDs were more than 5 times more likely to delay or forgo counseling due to cost, despite high reliance on public insurance. This pattern underscores system-level constraints, including narrow provider networks and inadequate reimbursement.^33^ Families of individuals with IDDs often experience significant out-of-pocket expenses for mental health services and are frequently unable to find clinicians who accept public insurance or have expertise in working with individuals with IDDs.^34^ To address these barriers, policies should prioritize Medicaid parity, increase reimbursement rates for mental health providers serving individuals with IDDs, and support coordinated care models that integrate case management and benefits navigation support. Innovative approaches, including exploring how AI might support inclusive care, should also be explored.^35^

Taken together, these findings underscore the need for a more deliberate and equity-oriented research agenda for adults with IDDs. Pervasive disparities in diagnosis, treatment, and outcomes are driven in part by the systematic exclusion of people with IDDs from clinical and health services research. Advancing equity in mental health will require inclusion of adults with IDDs in federally funded research, development and validation of IDD-specific mental health outcome measures, targeted investment in research addressing mental health conditions in this population, and stronger partnerships with advocacy organizations to ensure accessibility and relevance of study design.^32,36,37^ Additional priorities include training researchers and clinicians in disability-informed care, allocating resources for accommodations, and providing clearer guidance to institutional review boards to streamline inclusion. These reforms are essential to building an evidence base capable of informing disability-inclusive mental health care.

### Strengths and Limitations

A strength of this study is that it is among the few to use nationally representative data to quantify mental health disparities and access barriers among US adults with IDDs. However, the study also has several limitations that warrant consideration. First, adults with IDDs were identified using self- or proxy-reported functional limitations based on the WG-SS rather than clinical diagnoses. Although this approach aligns with population-based surveillance practices, it may misclassify some individuals, underrepresent adults with milder or later-identified disabilities, and overrepresent those with more severe functional limitations, thereby limiting generalizability. In addition, the use of broad functional criteria precluded analyses by specific diagnostic subgroups (eg, autism spectrum disorder), limiting insight into heterogeneity within the IDD population. Reliably identifying adults with IDDs in national administrative and survey datasets remains a persistent challenge.^38^ Second, all mental health outcomes were based on self-report or proxy report and may be influenced by recall bias, stigma, communication differences, or cognitive limitations. Third, the comparison group consisted of adults without functional limitations and differed from the IDD group in age and socioeconomic characteristics. To mitigate potential confounding, all analyses adjusted for key demographic and socioeconomic covariates, and sensitivity analyses using multiple imputation and propensity score weighting yielded results consistent with primary findings. Last, the cross-sectional design precludes causal inference.

## Conclusions

The findings of this cross-sectional study suggest that US adults with IDDs have higher rates of mental health conditions, treatment use, and cost-related barriers compared with those without functional impairments. The persistent disparities in mental health access for adults with IDDs reflect a broader systemic failure to include this population in mental health policy, research, and service infrastructure.^32^ Adults with IDDs remain significantly underrepresented in public health surveillance and are routinely underserved by conventional behavioral health systems.^7^ Advancing equity in care will require structural reforms, including integrating disability status into national datasets, improving the cognitive accessibility of mental health programs, and sustained investment in inclusive therapies and support. Importantly, efforts to improve the design of accessible surveys, interventions, and supports must center the voices of people with IDDs and their communities through community-engaged research and service development.^32,36^ These disparities reflect areas for needed improvement in public health surveillance, service design, and policy implementation, and come at a time when public health financing of health care is being cut.^39,40^ Addressing these inequities will require a sustained commitment to disability-inclusive health policy, investment in accessible and affordable mental health services, and collaboration between systems that support people with IDDs.

## Notes

Supplement 1. **eTable 1.** Prevalence and Adjusted Odds Ratio (AOR) of Anxiety Disorder or Depression Diagnosis, Pharmacologic Treatments, Frequency and Severity of Symptoms, and Use of and Access to Mental Health Services **eTable 2.** Summary of Responses to Relevant WG-SS Questions Among Adults With IDDs (n = 796) **eTable 3.** Adjusted Odds Ratio (AOR) of Anxiety Disorder or Depression Diagnosis, Pharmacologic Treatments, Frequency and Severity of Symptoms, and Use of and Access to Mental Health Services Among Noninstitutionalized Adults Aged 22-85 Across Survey Years (2021-2023)

Supplement 2.
Data Sharing Statement
