# Supplementary material for: Anxiety, Depression, and Care Barriers in Adults With Intellectual and Developmental Disabilities
Source: JAMA Netw Open. 2026 Feb 20;9(2):e2560205. doi: 10.1001/jamanetworkopen.2025.60205 (PMC12924096; doi:10.1001/jamanetworkopen.2025.60205)
Supplement: Supplement 1. — eTable 1. Prevalence and Adjusted Odds Ratio (AOR) of Anxiety Disorder or Depression Diagnosis, Pharmacologic Treatments, Frequency and Severity of Symptoms, and Use of and Access to Mental Health Services eTable 2. Summary of Responses to Relevant WG-SS Questions Among Adults With IDDs (n = 796) eTable 3. Adjusted Odds Ratio (AOR) of Anxiety Disorder or Depression Diagnosis, Pharmacologic Treatments, Frequency and Severity of Symptoms, and Use of and Access to Mental Health Services Among Noninstitutionalized Adults Aged 22-85 Across Survey Years (2021-2023) [file jamanetwopen-e2560205-s001.pdf]

Supplementary Online Content

Osuna AR, Kennedy J, Zhou C, Christakis DA. Anxiety, depression, and care barriers in adults with intellectual and developmental disabilities. *JAMA Netw Open.* 2026;9(2):e2560205. doi:10.1001/jamanetworkopen.2025.60205

eTable 1. Prevalence and Adjusted Odds Ratio (AOR) of Anxiety Disorder or Depression Diagnosis, Pharmacologic Treatments, Frequency and Severity of Symptoms, and Use of and Access to Mental Health Services

eTable 2. Summary of Responses to Relevant WG-SS Questions Among Adults With IDDs (n = 796)

eTable 3. Adjusted Odds Ratio (AOR) of Anxiety Disorder or Depression Diagnosis, Pharmacologic Treatments, Frequency and Severity of Symptoms, and Use of and Access to Mental Health Services Among Noninstitutionalized Adults Aged 22-85 Across Survey Years (2021-2023)

This supplementary material has been provided by the authors to give readers additional information about their work.

© 2026 Osuna AR et al. *JAMA Network Open*.

eTable 1. Prevalence and Adjusted Odds Ratio (AOR) of Anxiety Disorder or Depression Diagnosis, Pharmacologic Treatments, Frequency and Severity of Symptoms, and Use of and Access to Mental Health Services

**Mental health variables**	**IDD vs. No limitations**	
	aOR (95% CI)	p-value
**Anxiety**		
Diagnosed with anxiety disorder	8.91 (6.78, 11.71)	<.001
Taking medication for anxiety	6.65 (5.00, 8.85)	<.001
Feeling anxious daily	10.30 (7.66, 13.84)	<.001
Feeling a lot of anxiety	6.50 (4.95, 8.54)	<.001
**Depression**		
Diagnosed with depression	9.72 (7.35, 12.85)	<.001
Taking medication for depression	8.62 (6.56, 11.32)	<.001
Feeling depressed daily	17.00 (12.42, 23.28)	<.001
Feeling a lot of depression	8.43 (6.55, 10.86)	<.001
**Mental health services**		
Received counseling or therapy in past 12 months	6.26 (4.80, 8.17)	<.001
Needed, but delayed, counseling or therapy due to cost	6.53 (4.83, 8.82)	<.001
Needed, but did not get, counseling or therapy due to cost	7.90 (5.91, 10.55)	<.001

Respondents include non-institutionalized adults aged 22-85 with IDDs and adults without functional limitations, with analysis ***based on Propensity Score weighting and Multiple Imputations.*** Using multivariate imputations with chained equations (MICE), we generated 5 imputed datasets. For each imputed dataset, we conducted propensity score weighting (PSW) analysis to balance the following covariates: age, sex, race/ethnicity, region, community population size, income, insurance, household education, and marital status. Next, we updated the survey sampling weights with the propensity score weights and performed the weighted regression analysis with the updated sampling weights. Finally, we combined the results across the 5 imputed datasets using Rubin's rules. The results are listed in the Supplemental Table 1. There were some changes in effect sizes, but all conclusions from available data analysis remained unchanged. There was only minor impact from missing data.

© 2026 Osuna AR et al. *JAMA Network Open*.

eTable 2. Summary of Responses to Relevant WG-SS Questions Among Adults With IDDs (n = 796)

Variable	Unweighted frequency (%)	Weighted frequency in millions (%)
Difficulty communicating		
No difficulty	418 (52.58%)	4.03 (46.93%)
Some difficulty	165 (20.75%)	1.86 (21.64%)
A lot of difficulty	185 (23.27%)	2.33 (27.04%)
Cannot do at all	27 (3.40%)	0.38 (4.40%)
(Missing)	1	
Difficulty remembering/concentrating		
No difficulty	80 (10.11%)	0.98 (11.53%)
Some difficulty	83 (10.49%)	0.96 (11.22%)
A lot of difficulty	611 (77.24%)	6.39 (74.82%)
Cannot do at all	17 (2.15%)	0.21 (2.43%)
(Missing)	5	
Difficulty with self-care		
No difficulty	526 (66.08%)	5.32 (61.73%)
Some difficulty	112 (14.07%)	1.27 (14.74%)
A lot of difficulty	94 (11.81%)	1.12 (13.04%)
Cannot do at all	64 (8.04%)	0.90 (10.49%)

© 2026 Osuna AR et al. *JAMA Network Open*.

eTable 3. Adjusted Odds Ratio (AOR) of Anxiety Disorder or Depression Diagnosis, Pharmacologic Treatments, Frequency and Severity of Symptoms, and Use of and Access to Mental Health Services Among Noninstitutionalized Adults Aged 22-85 Across Survey Years (2021-2023)

**Mental health variables**	**2022 vs. 2021**		**2023 vs. 2021**	
	aOR (95% CI)	p-value	aOR (95% CI)	p-value
**Anxiety**				
Diagnosed with anxiety disorder	1.09 (0.99, 1.19)	.07	1.11 (1.02, 1.20)	**.018**
Taking medication for anxiety	1.16 (1.06, 1.27)	**.002**	1.09 (1.00, 1.20)	.06
Feeling anxious daily	1.05 (0.95, 1.17)	.34	1.10 (1.00, 1.21)	.06
Feeling a lot of anxiety	1.03 (0.92, 1.16)	.61	1.03 (0.91, 1.16)	.67
**Depression**				
Diagnosed with depression	1.02 (0.92, 1.12)	.72	1.12 (1.03, 1.23)	**.009**
Taking medication for depression	1.11 (1.00, 1.24)	.06	1.09 (0.99, 1.22)	.09
Feeling depressed daily	1.01 (0.83, 1.24)	.89	0.92 (0.75, 1.13)	.42
Feeling a lot of depression	1.04 (0.89, 1.22)	.59	1.10 (0.95, 1.29)	.21
**Mental health services**				
Received counseling or therapy in past 12 months	1.14 (1.03, 1.26)	**.009**	1.20 (1.09, 1.31)	**<.001**
Needed, but delayed, counseling or therapy due to cost	1.34 (1.15, 1.58)	**<.001**	1.38 (1.19, 1.60)	**<.001**
Needed, but did not get, counseling or therapy due to cost	1.30 (1.11, 1.53)	**.001**	1.40 (1.20, 1.62)	**<.001**

Based on the same models for Table 2 in the main manuscript.

© 2026 Osuna AR et al. *JAMA Network Open*.
