# Supplementary material for: Anxiety, Depression, and Care Barriers in Adults With Intellectual and Developmental Disabilities
Source: JAMA Netw Open. 2026 Feb 20;9(2):e2560205. doi: 10.1001/jamanetworkopen.2025.60205 (PMC12924096; doi:10.1001/jamanetworkopen.2025.60205)
Supplement: Supplement 2. — Data Sharing Statement [file jamanetwopen-e2560205-s002.pdf]

Data Sharing Statement

Osuna. Anxiety, Depression, and Care Barriers in Adults With Intellectual and Developmental Disabilities. *JAMA Netw Open*. Published February 20, 2026.
doi:10.1001/jamanetworkopen.2025.60205

Data
Data available: No
